# Evaluation of Primary Healthcare Centers’ Service Availability and Readiness for Implementing Diabetes Care in Abuja, Nigeria: A Cross-Sectional, Formative Assessment

**DOI:** 10.21203/rs.3.rs-3959541/v1

**Published:** 2024-03-26

**Authors:** Ikechukwu A. Orji, Abigail S. Baldridge, Mercy U. Ikechukwu-Orji, Bolanle Banigbe, Nelson C. Eze, Aashima Chopra, Kasarachi Omitiran, Guhan Iyer, Deborah Odoh, Morenike Alex-Okoh, Rifkatu Reng, Lisa R. Hirschhorn, Mark D. Huffman, Dike B. Ojji

**Affiliations:** Cardiovascular Research Unit, University of Abuja Teaching Hospital, Gwagwalada, Abuja; Department of Medical Social Science, Northwestern University Feinberg School of Medicine and Robert J Havey Institute for Global Health, Feinberg School of Medicine, Chicago, Illinois; Cardiovascular Research Unit, University of Abuja Teaching Hospital, Gwagwalada, Abuja; Resolve to Save Lives, New York; Department of Public Health, Federal Ministry of Health, Abuja; Department of Medical Social Science, Northwestern University Feinberg School of Medicine and Robert J Havey Institute for Global Health, Feinberg School of Medicine, Chicago, Illinois; Cardiovascular Research Unit, University of Abuja Teaching Hospital, Gwagwalada, Abuja; Cardiovascular Division and Global Health Center, Washington University in St. Louis, St. Louis, Missouri; Department of Public Health, Federal Ministry of Health, Abuja; Department of Public Health, Federal Ministry of Health, Abuja; Prof. F. Anuma Diabetes & Endocrine Center, University of Abuja Teaching Hospital, Gwagwalada, Abuja; Department of Medical Social Science, Northwestern University Feinberg School of Medicine and Robert J Havey Institute for Global Health, Feinberg School of Medicine, Chicago, Illinois; Cardiovascular Division and Global Health Center, Washington University in St. Louis, St. Louis, Missouri; Cardiovascular Research Unit, University of Abuja Teaching Hospital, Gwagwalada, Abuja

**Keywords:** service availability, service readiness, primary healthcare centers, diabetes care, Abuja, Nigeria

## Abstract

**Introduction:**

Noncommunicable diseases (NCDs) are associated with a high and rising burden of morbidity and mortality in sub-Saharan Africa, including Nigeria. Diabetes mellitus (DM) is among the leading causes of NCD-related deaths worldwide and is a foremost public health problem in Nigeria. As part of the National Multi-Sectoral Action Plan for the Prevention and Control of NCDs, Nigeria has committed to implementing the World Health Organization (WHO) Package of Essential NCD control interventions. Implementing the intervention requires the availability of essential elements, including guidelines, trained staff, health management information systems, equipment, and medications, in primary healthcare centers (PHCs). This study assessed the availability of the WHO package components and the readiness of PHCs to implement a DM screening, evaluation, and management program.

**Methods:**

This cross-sectional formative assessment adapted the WHO Service Availability and Readiness Assessment (SARA) tool to survey 30 PHCs selected by multistage sampling for readiness to deliver DM diagnosis and care in Abuja, Nigeria, between August 2021 and October 2021. The service availability and readiness indicator scores were calculated based on the proportion of PHCs with available DM care services, minimum staff requirement, diagnostic tests, equipment, medications, and national guidelines/protocols for DM care within the defined SARA domain.

**Results:**

All 30 PHCs reported the availability of at least two full-time staff (median [interquartile range] staff = 5 [4–9]), which were mostly community health extension workers (median [interquartile range]) = 3 [1–4]. At least one staff member was recently trained in DM care in only 11 (36%) of the PHCs. The study also reported high availability (100%) of paper-based health management information systems (HMIS) and DM screening services using a glucometer (87%), but low availability of DM treatment (23%), printed job aids (27%), and national guidelines/protocols (0%).

**Conclusion:**

This systematic assessment of PHCs’ readiness to implement a DM screening, evaluation, and management program in Abuja demonstrated readiness to integrate DM care into PHCs in terms of equipment, paper-based HMIS, and nonphysician health workers’ availability. However, strategies are needed to promote DM health workforce training, provide DM management guidelines, and ensure a reliable supply of essential DM medications.

## BACKGROUND

Noncommunicable diseases (NCDs, including diabetes mellitus (DM), are collectively responsible for almost three-quarters (75%) of global deaths, and most of these deaths (86%) occur in low- and middle-income countries.^1^ Moreover, cardiovascular diseases, cancers, chronic respiratory disease, and DM account for more than 80% of the global premature NCD mortality (i.e., deaths between 30 and 69 years).^2^ In Nigeria, nearly 30% of all deaths and 22% of premature mortality are due collectively to NCDs, which include DM.^1^ An estimated 3.6 million adults in Nigeria were affected by DM in 2021,^3^ and a recent study reported a progressive increase in the prevalence of DM in Nigeria over the past three decades.^4^ This figure is projected to increase if a concerted effort does not stem the tide. Therefore, as part of the national response to the increasing NCD burden, including DM, the Federal Ministry of Health has planned to implement the World Health Organization (WHO)-recommended package of essential NCD interventions,^5^ at all primary healthcare centers (PHCs) as part of the National Multi-Sectorial Action Plan (NMSAP) for the prevention and control of NCDs.^6^

This package of essential NCD interventions includes cost-effective activities aimed at the early detection, treatment, and control of DM and other NCDs to prevent life-threatening complications.^5^ However, the effective implementation of these interventions requires that health facilities have the necessary inputs, including guidelines, trained staff, equipment, and medications.^7,8^ Additionally, facilities must have functional health management information systems (HMIS) to document patient information and track disease control status and retention in care. There is a dearth of information on the availability of these inputs at PHCs in Nigeria, which is a prerequisite for any meaningful implementation, sustainment, or scale-up.

This study, therefore, aims to fill this gap by assessing readiness and service availability for integrating DM screening, evaluation, and management services into PHCs in Abuja, Nigeria, leveraging the existing program infrastructure of the ongoing Hypertension Treatment in Nigeria (HTN) Program (NCT0415815411).^9^ The ultimate goal is to contextualize, implement, and evaluate strategies to address gaps and improve the delivery of an evidence-based intervention known to reduce DM-related morbidity and mortality by improving screening, diagnosis, treatment, and control of DM. Thereby strengthening and positioning the PHCs toward achieving the objectives of the NMSAP for NCD prevention and control.^6^

## Objective

The study aimed to evaluate the capacity and readiness of health facilities for DM screening, diagnosis, and treatment at PHCs participating in the HTN Program. The team assessed (1) DM service availability at the PHC level, (2) specific service readiness for DM diagnosis and treatment, and (3) the availability of staff, job aids for DM care, HMIS, equipment, and medications for DM screening and treatment.

## METHODS

### Study Design

The study was a cross-sectional, formative assessment.

### Setting

The study was conducted from August 2021 to October 2021 in 30 PHCs in Abuja, the Federal Capital Territory (FCT), Nigeria. These sites were selected from the 60 PHCs participating in the Hypertension Treatment in Nigeria (HTN) Program. The HTN Program PHCs were originally sampled through a multistage sampling technique and achieved an even spread across the FCT.^10^ Abuja the study location, is the administrative capital of Nigeria and is located in the north-central geo-political zone of Nigeria.^11^ It has six area councils (the equivalent of local government areas) with 62 political wards and more than 243 PHCs.^10^ The HTN Program is an ongoing National Heart, Lung, and Blood Institute-funded type II hybrid implementation-effectiveness study of 60 selected PHCs in Abuja. The HTN Program began recruiting patients in January 2020 and aimed to improve awareness, diagnosis, treatment, and control of hypertension in Abuja, the Federal Capital Territory of Nigeria.^9^

### Site Selection

The 30 PHCs were selected by random sampling using a computer-based statistical program across the six area councils. The process of sampling included the following steps: (1) identifying HTN Program PHCs (n = 60); (2) excluding the security-challenged sites (n = 14); and (3) sampling 50% of the original number of HTN PHCs in each area council to achieve the target sample size (n = 30). The team contacted each selected PHC to ascertain their willingness to participate in this formative survey and received a positive affirmation from all eligible PHCs. The study team visited each PHC facility, obtained informed consent, and conducted the survey on the appointed days.

### Survey Adaptation

The study team adapted the WHO Service Availability & Readiness Assessment (SARA) tool^12^ and used it to assess the availability and readiness for diabetes care in the selected PHCs (Appendix A: additional file 1). The WHO defines service readiness as the capability of health facilities to offer a specific service, as measured through selected indicators, including trained staff, printed guidelines, essential equipment, diagnostic capacity, and required medications and commodities.^12^ This study assessed the presence or absence of each of the following SARA indicators: (1) staffing and training, which refers to at least one staff member being trained in diabetes care within the last 24 months. (2) DM service availability, (3) job aid for DM care service delivery, including national guidelines for the prevention, diagnosis, and management of DM and other guidelines, (4) health management information system, (5) DM screening equipment and supplies, and (6) essential DM medications. The team completed the survey using the adapted SARA tool (Appendix A; additional file 1) and the observation checklist (Appendix B; additional file 2).

These indicators are described below:

1. Staffing and training
  Staffing refers to the availability or otherwise of two or more full-time health workers as adequate, the benchmark used in the HTN Program,^10^ and includes the category of nonphysician health workers (community health extension workers and nursing professionals cadres) who are qualified to prescribe diabetes medications under the Nigerian National Task-shifting and Task-sharing (NTSTS) policy for the Prevention and Control of NCDs in Nigeria, recently approved for use by the Federal Ministry of Health and Social Welfare to adopt^13,14^. The training component was measured by the proportion of PHCs with at least one staff member trained in diabetes care within the last 24 months.
2. DM service availability.
  DM service availability was defined as the availability of diabetes mellitus treatment and/or diagnostic services at the PHC facility during the time of the survey.
3. Job aid for DM care service delivery, including national guidelines and other for the prevention, diagnosis, and management of DM.
  This indicator refers to the availability or otherwise at the PHC of job aids for DM care, such as the national guidelines and other related guidelines for the Prevention, Diagnosis, and Management of DM; printed checklists for screening patients for risk of diabetes; printed checklists for interventions for patients with risk factors for cardiovascular diseases (CVDs) and DM; and cardiovascular risk assessment charts.
4. Health management information system.
  The health management information system refers to the availability or otherwise of electronic and/or paper-based medical records for all patients, and patients with NCDs. These data comprised records of patient visits, patient record files, and individual records for patients with NCDs, including DM, with usability for longitudinal monitoring of patient care.
5. DM screening equipment and supplies.
  DM screening equipment and supplies were defined as the availability or otherwise of a functional point-of-care glucometer and valid glucometer strip, according to the recommendations of the Nigerian NTSTS policy for the prevention and control of NCDs at the primary healthcare level in the country.^13,14^
6. Essential DM medications.
  Essential DM medications describe the availability of at least a 30-day stock of 500 mg metformin and 5 mg glibenclamide. These two medications are among those approved for use in the Nigerian national guidelines on the prevention, control, and management of DM.^15^

**Diabetes Service Readiness** reflects the availability of the essential indicators for diabetes care at the PHCs, including the availability of two or more full-time staff approved to prescribe diabetes medications, treatment, and/or provide diagnostic services; a functional glucometer and valid test strip; DM medications of at least a 30-day dose of glibenclamide and metformin; the availability of national guidelines on the prevention, diagnosis, and management of DM; and the availability of health management information systems that can be used for longitudinal monitoring of patients.

### Data collection process

The research team completed the SARA surveys by interviewing the most senior clinical staff members available at each PHC on the day of the visit after providing written informed consent. These staff members included facility managers and heads of units from maternity, laboratory, and pharmacy units. The team visited the various units to observe the availability of self-reported materials, including the pharmacy and the laboratory sections, to confirm the availability of DM medications and diagnostic equipment, respectively. On the day of the visit, the study team used a checklist (Appendix B; Additional file 2) to document the presence of a functional glucometer, test strips, and selected DM medications, namely, metformin and glibenclamide, which are among the medications approved for use in the national guideline,^15^ and listed in the Nigeria Essential Medicine List 2020, 7th Edition for the management of DM.^16^

### Statistical analyses

The results of the facility-based evaluation of DM care service availability and readiness were tabulated. Continuous variables are summarized as the mean and standard deviation, and nonparametrically distributed variables are summarized as the median and interquartile range. Categorical results are reported as frequencies. The domains of interest to the research team were based on the availability and readiness of the PHCs to deliver DM care, including staffing and training, DM service delivery, health management information systems, equipment for DM screening, and medications for DM treatment. The service availability and readiness indicator scores were calculated based on the proportion of PHCs with available DM care services, minimum personnel requirement, diagnostic tests, equipment, medications, and national guidelines/protocols for diabetes care within the defined SARA domain question bank. The study team used R version 3.5.1 (R Foundation, Vienna, Austria) and Microsoft Excel version 2016 (Microsoft, Redmond, Washington) for statistical analysis.

## RESULTS

### Participants

**Figure 1:**
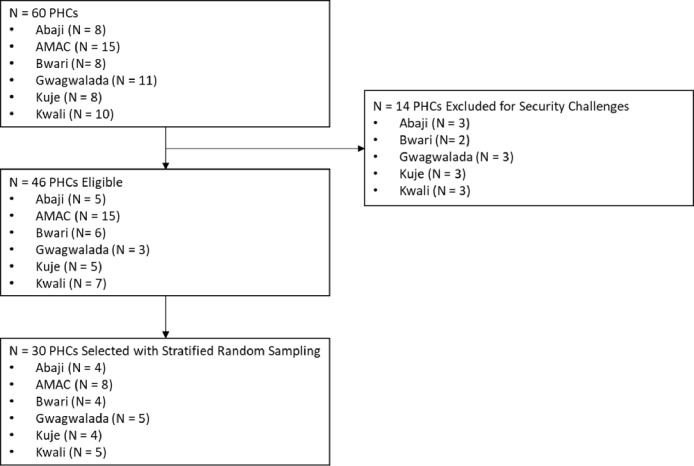
Site selection flow chart. AMAC=Abuja Municipal Area Council; FCT=Federal Capital Territory; PHCs=primary healthcare centers

The flowchart of the assessed sites is shown in Fig. 1 below. The SARA assessment was completed for all 30 selected PHCs.

### Definitions of the key indicators

Table 1 below, provides definitions of the key indicators.

### Staffing and training

Table 2 summarizes the service availability and readiness assessment for diabetes care integration across the included domains. Among all (n = 30, 100%) participating PHCs, there were at least two full-time staff at the time of survey administration. The median [interquartile range] number of full-time staff was 5 [4–9]; most were community health extension workers, with a median [interquartile range] = 3 [1–4], followed by nurses (median [interquartile range] = 1 [0–2]). In terms of personnel training, 11 (37%) of the sites reported at least one staff member trained in the diagnosis and management of diabetes within the last two years.

### DM service and job aid availability

Among the 30 PHCs surveyed, 26 (87%) could provide on-site DM diagnosis, while 6 (20%) sites reported on-site capacity for DM treatment. The national guidelines or protocols for the diagnosis and management of DM at primary health centers and cardiovascular risk assessment were absent in all the surveyed facilities (n = 0; 0%). There was also a low availability of other job aids, such as job aids for screening patients for risk of DM (n = 8; 27%), job aids for interventions for patients with risk factors for CVDs and DM (n = 7; 23%), and other guidelines/protocols for the diagnosis and management of DM (n = 8; 27%), [Table 2].

### Health management information systems

No (n = 0; 0%) PHCs reported having an electronic medical records system; however, all (n = 30; 100%) PHCs reported the availability of a paper-based health management information system, including records of patient visits, maintaining patient files for all their patients, and individual records for patients with NCDs [Table 2].

### DM service readiness

Table 3 presents diabetes service readiness indicators for the surveyed PHCs in the six area councils of Abuja. Diabetes service readiness reflects the composite availability of the essential indicators for DM care at the PHCs presented according to the area councils’ performance. The staffing indicator revealed adequate staff for DM service in all the area councils in the FCT, Abuja (100%). This indicator refers to the proportion of facilities with two or more full-time nonphysician health workers approved to prescribe diabetes medication according to the recommendations of the Nigerian NCD TSTS policy guideline. The equipment indicator, defined as the proportion of facilities with available functional glucometers and valid test strips for point-of-care screening for DM, varied from 60–100% across the area councils and an average of 87% for FCT, Abuja. The medication availability indicator refers to the proportion of PHCs across the area councils with at least a 30-day dose of 500 mg metformin and 5 mg glibenclamide found in the PHC pharmacy inventory and drug shelf or cabinet on the day of the assessment. This indicator was a 3% score as just one PHC in the Bwari area council had the requisite DM medication. The National guideline indicator score which refers to the proportion of facilities with available national guidelines/protocols for diagnosing and treating DM was 0% across the area council. The paper-based HMIS, defined as the proportion of PHCs with paper-based medical records for all patients, including patients with NCDs, and usable for longitudinal patient monitoring, is 100% in all the 30 PHCs assessed.

## DISCUSSION

In line with the WHO’s target to reduce premature death from NCDs, including diabetes, by 30% by 2030, Nigeria launched the National Multi-Sectorial Action Plan for the Prevention and Control of NCDs in 2019, including its plan to implement the WHO Package of Essential NCD interventions at all PHCs.^17,18^ Although there are ongoing system-level hypertension control programs at the primary health care level, such as the HTN Program in 60 PHCs in Abuja^19^, no such programs have yet been implemented for DM. Across the surveyed 30 PHCs in the HTN Program, DM care service availability and readiness were high in the health workforce and paper-based health management information systems. The current study also revealed variability in equipment availability and low scores in the availability of diabetes medicines and national guidelines or protocols. The survey demonstrated the readiness to integrate diabetes care into these PHCs in terms of the availability of paper-based health management information systems, equipment, and personnel (nonphysician health workers). However, the strategy to use nonphysician health workers requires an investment in diabetes care training and longitudinal retraining in alignment with the provisions of the NTSTS policy for the prevention and control of NCDs in Nigeria.^13,14^ The findings also revealed the need to provide access to DM guidelines and protocols and supply chain strengthening to ensure a reliable supply of quality DM medications and equipment.

### Staffing and training

The study reported adequate nonphysician full-time staff among all PHCs surveyed, with a cut-off of two or more full-time staff used to define adequacy for diabetes service delivery, the same criterion used in the HTN Program.^12^ The staffing component is focused on the category of nonphysician health workers (community health extension workers (CHEWs) and nursing professional cadres). approved to prescribe diabetes medications according to the recommendation of the National Task-Shifting/Task Sharing policy for the prevention and control of NCDs^13,14^

The findings showed a median of three full-time CHEWs across the surveyed sites, which aligns with the minimum number of three CHEWs recommended for PHC by the Nigerian National Primary Health Care Development Agency.^20^ This finding is similar to that of a recent PHC facility assessment survey in different states in Nigeria, which revealed that CHEWs were the most common cadres of staff across the PHCs surveyed,^21,22^ and a 2017 WHO publication which reported the CHEWs as the largest cadre of health workforce in PHCs across Nigeria.^23^ This pattern may be partly attributed to the high availability of schools of health technology responsible for their training and the shorter duration of training, which has enabled a high turnover of CHEWs in Nigeria.^24^ In addition, the CHEW cadre was created to fill the health workforce gap in PHCs when it was established as the basic health unit in Nigeria in the late 1970s.^25^ Furthermore, there is a seeming preference for the hiring of CHEWs in the primary health care system over nurses/midwives by local government authorities in Nigeria as this appears more economically viable due lower salaries of the former.^26^ Our result demonstrates the high rating of staff availability for a nonphysician-led DM care service delivery. On the contrary, a significant gap in staff training for diagnosing and treating DM across the sites was identified, similar to the findings of a previous survey in Nigeria in which inadequate staff training was reported for managing HIV and tuberculosis coinfections, including multidrug-resistant tuberculosis;^27^ for CVD and hypertension management.^11^ Findings from other sub-Saharan African country reports also showed that the availability of trained staff was the poorest performing domain of diabetes service readiness.^28,29,30^ On the other hand a 2020 Nigerian study revealed the high availability of trained staff in the management of HIV and malaria, as well as immunization and family planning services.^21^ The low availability of staff trained in DM can be attributable to the previous task-shifting policy for essential services in Nigeria which focused on the management of HIV, malaria, tuberculosis, reproductive health, and maternal and newborn health and not on CVD.^26^ However, with the Nigeria National Task-Shifting/Task Sharing policy document of November 2023 now including treatment of CVD risk factors like DM and hypertension^13,14^ the availability of trained staff is likely to improve over time. The need for such improvement cannot be over-emphasized as the availability of qualified and trained staff remains an essential component in implementing the task-shifting/task-sharing strategy for NCD treatment, including DM.^31^ Therefore, bridging the training gap for these nonphysician health workers before initiating DM care integration, as well as longitudinal retraining, continuing medical education, and supportive supervision and mentoring, is required for successful DM care integration in PHCs in Nigeria.^32,33,34^

### Diabetes Service Availability and Readiness

Screening services and equipment which are glucometer and test strips for diagnosing DM were available in most of the PHCs surveyed, similar to the findings of a previous study at primary care facilities in Nigeria in which screening services for NCDs, including DM and hypertension, were available.^17^ Conversely, some studies have reported low availability of glucometers for DM screening in southwest Nigeria, at rural PHCs 33%^27^ and 46% at urban PHCs. In PHCs in Tanzania, an availability of 38%^23^ was reported while in Ethiopia 40% was reported.^24^ The high availability of DM screening services reported in this study is a facilitator for DM integration into these primary healthcare facilities. This finding may be due, at least in part, to support from the Federal Government PHC Revitalization Initiative through the ongoing Basic Health Care Provision Fund program implemented in some of the PHCs.^35^ Another reason for this finding may be due to the presence and activities of the Health Strategy and Delivery Foundation (HSDF), a nongovernmental organization that helped to implement diabetes screening programs in some of these PHCs in the Abuja Municipal Area Council and Bwari area councils between 2018 and 2021.^36^ Even though the availability of facilities for the screening of DM was high in these facilities, treatment services was low across all the 30 PHCs assessed, with only approximately one-fifth of sites reporting DM treatment service availability. This may be partly, due to the absence of policies and guidelines prior to this time which enabled non-physician healthcare workers to treat DM at the primary care level. Similarly, the medication indicator was very poor across all the 30 PHCs assessed with only one PHC having a 30-day stock of essential diabetes medications (i.e., metformin and glibenclamide), and this reflects low readiness for treatment. Our findings are similar to previous studies in PHCs in Nigeria that documented the absence of DM medication in all of the PHCs surveyed^37^ and low availability of blood pressure-lowering medications.^12,38^ The very low score in the DM medication indicator is partly attributed to the low availability of DM treatment services found among the PHCs surveyed. This is partly attributed to the lack of policies before this time that enabled non-physician healthcare workers to initiate treatment of DM at the primary care level, and the dearth of primary care physicians in these facilities. The low availability of DM medications represents an important, potentially modifiable barrier to high-quality DM care. There is an obvious need to contextualize and implement strategies to enhance diabetes medication accessibility. For example, a subsidized, drug-revolving fund mechanism could be developed to ensure a reliable supply of quality diabetes medications at PHCs with the provision of seed stock drugs, leveraging the experience from similar programs such as the HTN Program in Nigeria,^39^ and the Academic Model Providing Access To Healthcare (AMPATH) program in Kenya.^40^ However, for the long-term sustainability of diabetes medication accessibility, a social health financial protection plan, such as community-based health insurance, is recommended to reduce out-of-pocket expenditures related to DM treatment.^41^ This need was emphasized by stakeholders in a recent study that identified access to functional health insurance as a central strategy for accessing quality and affordable blood pressure-lowering medications.^39^ Furthermore, the national guidelines on the prevention, control, and management of DM were unavailable for all the PHCs surveyed. This finding represents a gap that needs to be addressed for a successful DM care integration at the primary care level. Even though, the National DM management guidelines of the Federal Ministry of Health of Nigeria,^15^ have been developed, they were not yet widely disseminated at the time of this survey. In addition, clinical desk guides and CHEW job aides for the management of NCDS at primary healthcare centers which are components of the newly approved policy document for the effective prevention and control of NCDs in Nigeria will be very useful in integrating DM into these primary care facilities.^13,14^

### Health Management Information Systems.

An adequate paper-based Health Management Information System was found across all 30 PHCs surveyed. This finding represents the availability of paper-based medical records of patients’ visits, patients’ files, and individual records for NCD patients and also available for longitudinal care in other conditions. However, electronic medical records were unavailable for any of the PHCs. While paper-based HMIS has been effective at the PHC level, it is challenging to use it for longitudinal follow-up and national coordination. Therefore, there is a need for a hybrid HMIS which includes a combination of paper-based and electronic-based health management information systems at the PHC level.^42^

A hybrid HMIS model piloted at the PHC level in Nigeria by the Resolve To Save Lives (a nongovernmental organization) comprising a paper-based HMIS and District Health Information System version 2 (DHIS2), an electronic HMIS, is recommended. DHIS2 software which is an open-source, web-based platform developed as a global collaboration and managed by the University of Oslo’s Health Information Systems Program (HISP) Center has been proven to be reliable for use as a health management information system.^43^ Such a hybrid paper-based DHIS2 electronic model piloted with Resolve To Save Lives holds great promise for improving timely reporting of quality NCD data directly from PHCs and can be used for prompt decision-making,^42^ which aligns with the country’s adoption of its use for the National Health Management Information System in 2010.^44^ Although it is not currently deployed at the PHC level, DHIS2 is implemented at the local government level in the Federal Capital Territory for the management of paper-based data generated at the health facility.

### Strengths and Limitations

The main strength of this study lies in the distribution of the facilities assessed throughout the six area councils of the Federal Capital Territory which makes generalizability throughout the FCT easy. Secondly, the use of the WHO SARA tool which is a global tool for primary health care facility assessment makes comparison of our findings with those of other studies possible. There are however some limitations, first, the study was a cross-sectional assessment using the adapted SARA instrument; therefore, the evaluation of temporal trends in service readiness and availability was beyond the scope of the current study. Secondly, the team limited the equipment and medication availability to a point-of-care glucometer, test strips, and a minimum supply of metformin and glibenclamide. Additional equipment like Hemoglobin A1c (HbA1c) testing devices and other medications may be desirable for screening/diagnosing and treating people with diabetes to meet the needs of the population served by these PHCs. Our assessment was however in keeping with recommended equipment and medications in the Nigerian national guidelines for diagnosing and treating diabetes at the PHCs.

## CONCLUSIONS

This was the first systematic assessment of the service availability and readiness of PHCs in the Federal Capital Territory, Abuja, Nigeria, for screening, diagnosing, and treating DM. The survey demonstrated readiness to integrate DM care into PHCs in terms of personnel in alignment with the national task-shifting policy, paper-based health management information systems, and equipment. However, strategies are needed to promote DM health workforce training, provide access to guidelines on the management of DM and simplified treatment protocols, and provide a reliable supply of essential DM medications. These can be successful as PHCs continue to leverage the national task-shifting/task-sharing strategy for the management of NCDs to enhance DM care integration into the PHCs.

## Figures and Tables

**Table 1:** Definitions of Key Indicators.

SN	Indicator	Definition
1.	Staff	The proportion of facilities with availability of two or more full-time nonphysician health workers approved to prescribe diabetes medication by the Nigerian NCD TSTS policy guideline.
2.	Diabetes service availability	The proportion of facilities offering diabetes mellitus treatment, and or diagnosis.
3.	Equipment	The proportion of facilities with the availability of a functional glucometer and valid test strip for point-of-care screening for diabetes. *Only the availability of a functional glucometer and valid glucometer strip assessed in line with the National guidelines for the prevention, diagnosis, and management of DM, for the basic equipment required at a PHC for diabetes diagnosis.
4.	Medications	The proportion of facilities with availability of at least one 30-day dose of metformin 500 mg and glibenclamide 5 mg. *Only the availability of metformin and glibenclamide assessed in line with National guidelines for the prevention, diagnosis, and management of DM for basic medication required at a PHC for diabetes treatment.
5.	National guideline/protocol	The proportion of PHCs with the availability of National guidelines for the prevention, diagnosis, and management of DM.
6.	Paper-based health management information system (HMIS)	The proportion of PHCs who maintain paper-based medical records for all patients, including patients with NCDs, that can be used for longitudinal follow-up of patients’ care.
7.	Diabetes Service Readiness	This is the availability of the essential indicators for diabetes care at the PHCs, including the availability; of two or more full-time staff approved to prescribe diabetes medications, treatment, and or diagnostic services, a functioning glucometer, and valid test strip, DM medications of at least a 30-day dose of glibenclamide and metformin, availability of and national guidelines for the prevention, diagnosis and management of DM, and the availability of health management information systems, that can be used for longitudinal monitoring of patients care.

**Table 2:** Service availability and readiness assessment for diabetes care integration at 30 PHCs in Abuja, Nigeria.

Site characteristics	Total No. Sites	Result
**Staffing and Training**		
Sites with two or more full-time staff,^a^ n (%)	30	30 (100)
Number of full-time healthcare professionals, median (IQR)	30	5 (4–9)
Full-time community health extension workers, median (IQR)	30	3 (1–4)
Full-time nurses, median (IQR)	30	1 (0–2)
Full-time doctors (generalists and specialists), median (IQR)	30	0 (0–0)
Received diabetes training within the past two years, n (%)	30	11 (37)
**Diabetes Service Availability**		
Diagnosis (On-site), n (%)	30	26 (87)
Treatment (On-site), n (%)	30	6 (20)
**Availability of Job Aids for Diabetes Care Service Delivery**		
National Guidelines for diagnosis and management, n (%)	30	0 (0)
Other Guidelines for diagnosis and management, n (%)	30	8 (27)
Printed checklist or job aid for screening patients for risk of diabetes, n (%)	30	8 (27)
Printed checklist/job aids for interventions for patients with risk factors for CVDs & DM, n %	30	7 (23)
Cardiovascular risk assessment charts, n (%)	30	0 (0)
**Health Management Information System**		
Facility keeps records of patients’ visits, n (%)	30	30 (100)
Facility keeps electronic patient files, n (%)	30	0 (0)
Facility keeps paper patient files, n (%)	30	30 (100)
Facility maintains electronic individual patient records for patients with NCDs, n (%)	30	0 (0)
Facility maintains paper individual patient records for patients with NCDs, n (%)	30	30 (100)

a Including all reported full-time doctors (generalists/specialists), nursing professionals, pharmacists, laboratory technicians, community health extension workers, and community health Officers. CVD: Cardiovascular Disease; IQR: Interquartile Range; NCDs: Noncommunicable Diseases

**Table 3:** Diabetes service readiness indicators of surveyed PHCs in the six area councils of FCT, Abuja.

Diabetes Care Service Indicator, No. (%)	(n = 4)	AMAC (n = 8)	Bwari (n = 4)	Gwagwalada (n = 5)	Kuje (n = 4)	Kwali (n = 5)	Total (n = 30)
Availability^a^	100%	88%	100%	100%	75%	60%	**87%**
Readiness Indicators^b^							
Staff^c^	4 (100%)	8 (100%)	4 (100%)	5 (100%)	4(100%)	5 (100%)	**30(100%)**
Equipment^d^*	4 (100%)	7 (88%)	4 (100%)	5 (100%)	3 (75%)	3 (60%)	**26 (87%)**
Medications^e^**	0 (0%)	0 (0%)	1 (25%)	0 (0%)	0 (0%)	0 (0%)	**1 (3%)**
National Guidelines/Protocols^f^	0 (0%)	0 (0%)	0 (0%)	0 (0%)	0 (0%)	0 (0%)	**0 (0%)**
Paper-based HMIS^g^	4 (100%)	8 (100%)	4 (100%)	5 (100%)	4(100%)	5 (100%)	**30 (100%)**

a Calculated as the proportion of facilities offering diabetes mellitus treatment and/or diagnosis.

b The diabetes service readiness reflects the proportion of PHCs with the essential indicators for diabetes care, including the availability of two or more full-time staff approved to prescribe diabetes medications, a functioning glucometer and valid test strip for DM screening and diagnostic services, DM medications of at least a 30-day dose of glibenclamide and metformin, availability of national guidelines or protocols for DM care, and the availability of paper-based health management information systems with potential for longitudinal patient monitoring.

c Calculated as the proportion of facilities with availability of two or more full-time nonphysician health workers approved to prescribe diabetes medication by the Nigerian NCD TSTS policy guideline.

d Calculated as the proportion of facilities with the availability of a functional glucometer and valid test strip for point-of-care screening for diabetes.

* Only the availability of a functional glucometer and valid glucometer strip assessed in line with the Nigerian National NCD guideline for basic equipment required at a PHC for diabetes diagnosis

e Calculated as the proportion of facilities with at least one 30-day dose of 500 mg metformin and 5 mg glibenclamide.

** Only the availability of metformin and glibenclamide assessed in line with National NCD guidelines for basic diabetes medication required at a PHC for diabetes treatment.

f Calculated as the proportion of facilities with available national guidelines/protocols for the diagnosis and treatment of diabetes mellitus.

g Calculated as the proportion of PHCs who maintain paper-based medical records for all patients, including patients with NCDs, and are used for longitudinal patient monitoring.

AMAC = Abuja Municipal Area Council, PHC = primary healthcare center

## Data Availability

The datasets used and/or analyzed during the current study are available from the corresponding author upon reasonable request.

## Abbreviations

AMAC: Abuja Municipal Area Council
AMPATH: Academic Model Providing Access To Healthcare
CVDs: Cardiovascular Diseases
CHEW: Community Health Extension Worker
DHIS2: District Health Information System version 2
DM: Diabetes Mellitus
FCT: Federal Capital Territory
HbA1C: Hemoglobin A1c
HISP: Health Information Systems Program
HIV: Human Immunodeficiency Virus
HMIS: Health Management Information System
HTN: Hypertension Treatment in Nigeria
IQR: Inter-Quartile Range
NCDs: Non-Communicable Diseases
NTSTS: National Task-Shifting and Task-Sharing
PH: Primary Healthcare Center
SARA: Service Availability and Readiness Assessment
WHO: World Health Organization
